# Supplementary material for: A pair-rule-like transcription network coordinates neural tube closure in a proto-vertebrate
Source: bioRxiv. 2025 Jun 25:2025.06.18.660479. Preprint. [Version 2] doi: 10.1101/2025.06.18.660479 (PMC12262576; doi:10.1101/2025.06.18.660479)
Supplement: Supplement 1 [file NIHPP2025.06.18.660479v2-supplement-1.pdf]

bioRxiv preprint doi: https://doi.org/10.1101/2025.06.18.660479; this version posted June 25, 2025. The copyright holder for this preprint (which was not certified by peer review) is the author/funder, who has granted bioRxiv a license to display the preprint in perpetuity. It is made available under aCC-BY 4.0 International license.

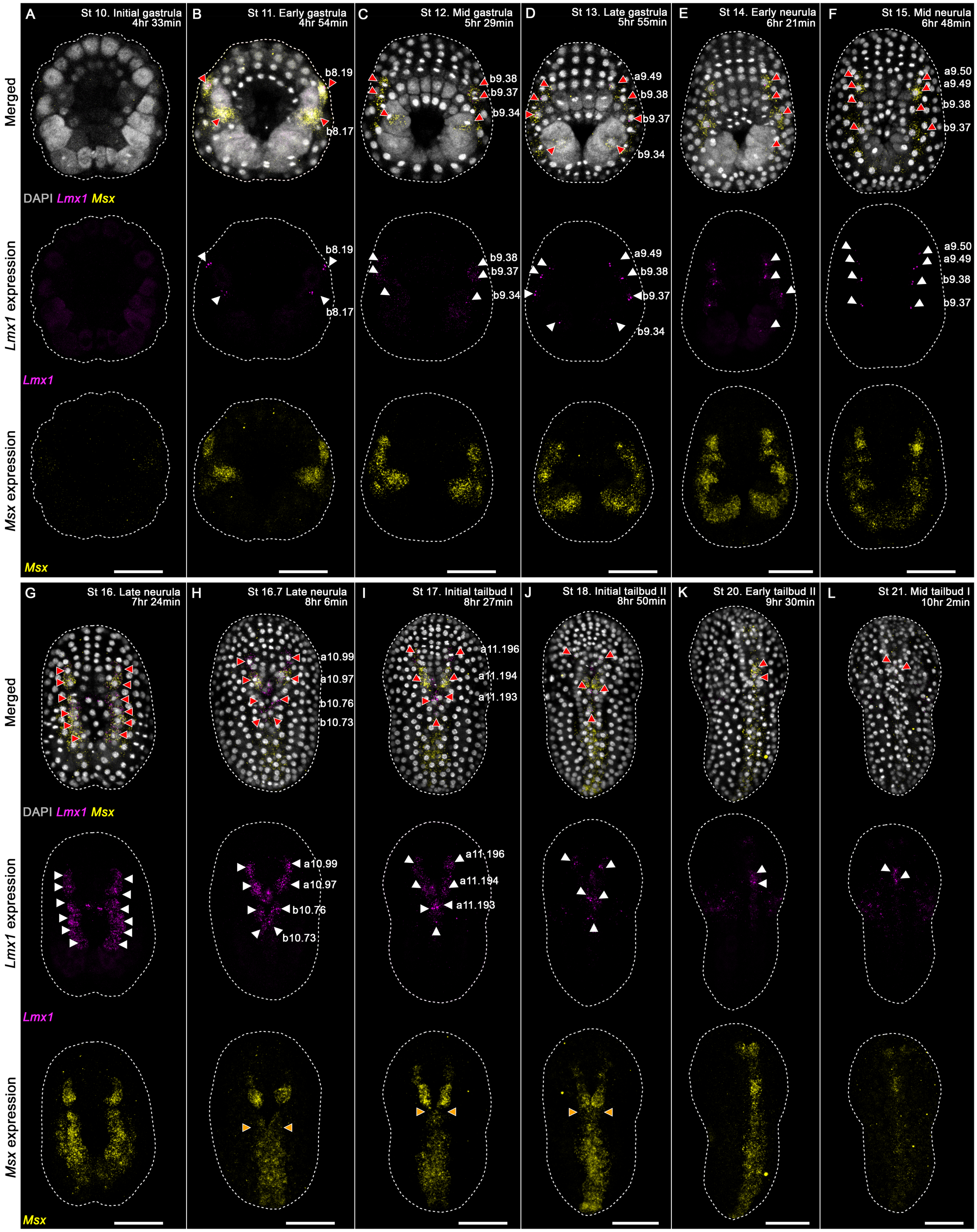

Figure S1: ***Lmx1*** **and** ***Msx*** **expression patterns throughout gastrulation and neurulation.** (A-L) Expression was examined throughout neurulation (∼4.5-10 hfp; samples collected every ∼20 minutes; 12 time points) via HCR *in situ* hybridization. The photographs are maximum-intensity projections of Z-projected image stacks overlaid in pseudocolor with HCR signals for *Lmx1* probe (magenta) and *Msx* probe (yellow). Nuclei were stained with DAPI (gray). Arrows (red/white) indicate *Lmx1* expression in descendants of neural plate border cells and neural plate cells, and (orange) *Msx* downregulation at zippering point. Brightness and contrast of images was adjusted linearly. Numbers of embryos examined n = 15 per time point over n = 5 experiments. Scale bars, 50 µm.

bioRxiv preprint doi: https://doi.org/10.1101/2025.06.18.660479; this version posted June 25, 2025. The copyright holder for this preprint (which was not certified by peer review) is the author/funder, who has granted bioRxiv a license to display the preprint in perpetuity. It is made available under aCC-BY 4.0 International license.

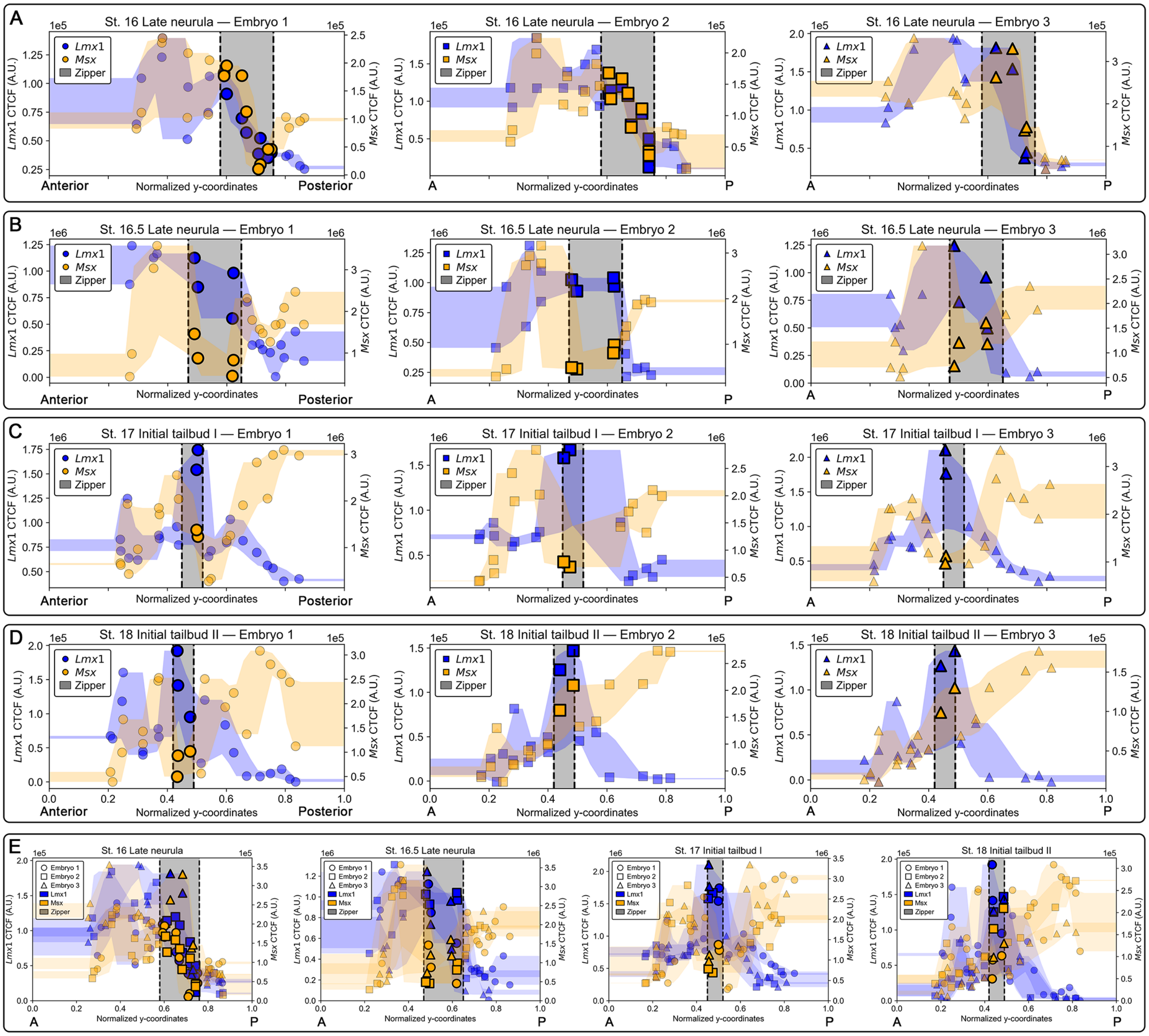

Figure S2: ***Lmx1* expression increases while *Msx* is selectively downregulated at the zippering point during neural tube closure.**

(A–D) Corrected total cell fluorescence (CTCF) for *Lmx1* (dark blue) and *Msx* (orange) HCR *in situ* hybridization signals in dorsal midline cells across four developmental stages of NTC, plotted by each cell's normalized y-coordinate (relative to the embryo height). Each point represents a single nucleus, and shaded regions denote the local range across neighboring points. Data are shown for n = 3 embryos per time point (n = 4), with marker shapes indicating individual embryos. During the Late Neurula stage (A), *Lmx1* and *Msx* are broadly co-expressed. As zippering initiates and progresses (B–C), *Lmx1* becomes locally enriched at the zippering point (gray region between dashed lines), while *Msx* becomes selectively downregulated in the same domain. (E) Aggregate plots from (A–D) illustrate consistency and embryo-to-embryo variability.

bioRxiv preprint doi: https://doi.org/10.1101/2025.06.18.660479; this version posted June 25, 2025. The copyright holder for this preprint (which was not certified by peer review) is the author/funder, who has granted bioRxiv a license to display the preprint in perpetuity. It is made available under aCC-BY 4.0 International license.

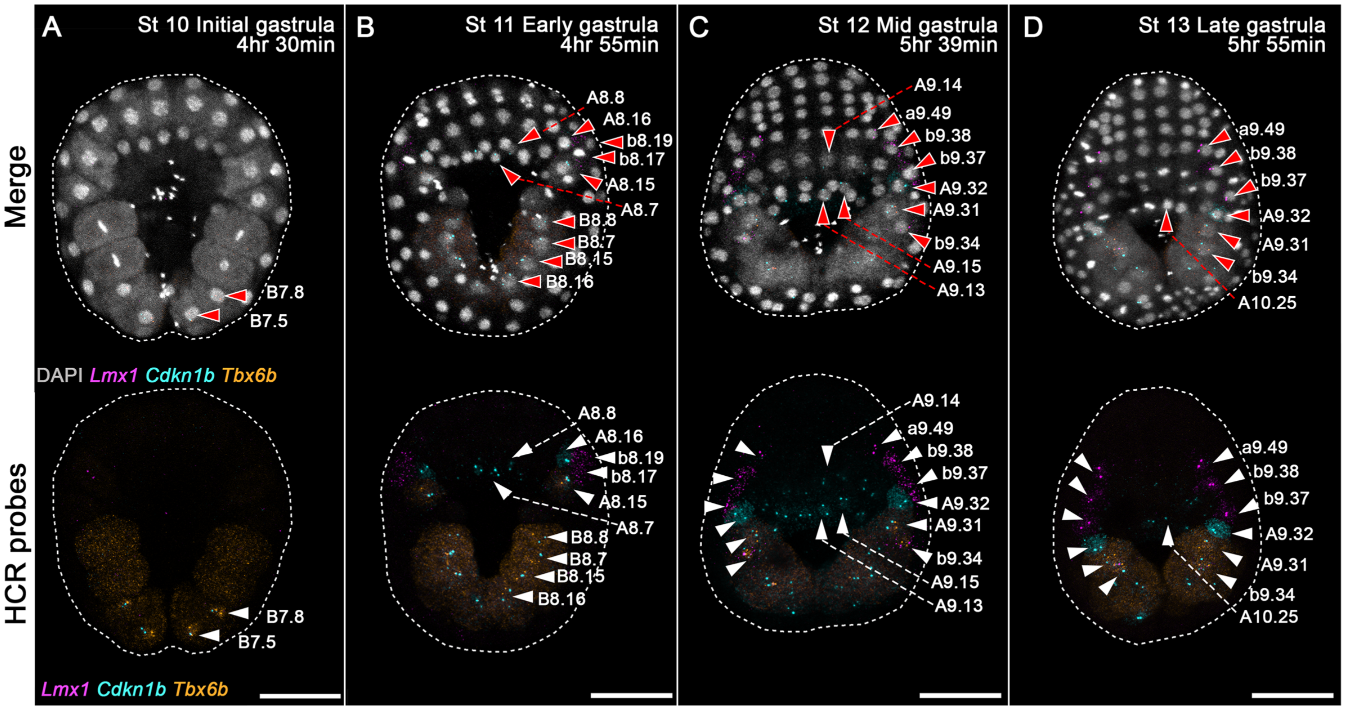

Figure S3: ***Lmx1*, *Cdkn1b*, and *Tbx6-b* expression patterns during gastrulation.**
(A-D) Expression was examined throughout gastrulation (∼4.5-6 hfp; samples collected every ∼30 minutes; four time points) via HCR *in situ* hybridization. The photographs are maximum-intensity projections of Z-projected image stacks overlaid in pseudocolor with HCR signals for *Lmx1* probe (magenta), *Tbx6-b* probe (orange), and *Cdkn1b* probe (cyan). Nuclei were stained with DAPI (gray). Developmental stages are indicated in the photographs. Brightness and contrast of images was adjusted linearly. Numbers of embryos examined n = 15 per time point over n = 3 experiments. Scale bars, 50 µm.

bioRxiv preprint doi: https://doi.org/10.1101/2025.06.18.660479; this version posted June 25, 2025. The copyright holder for this preprint (which was not certified by peer review) is the author/funder, who has granted bioRxiv a license to display the preprint in perpetuity. It is made available under aCC-BY 4.0 International license.

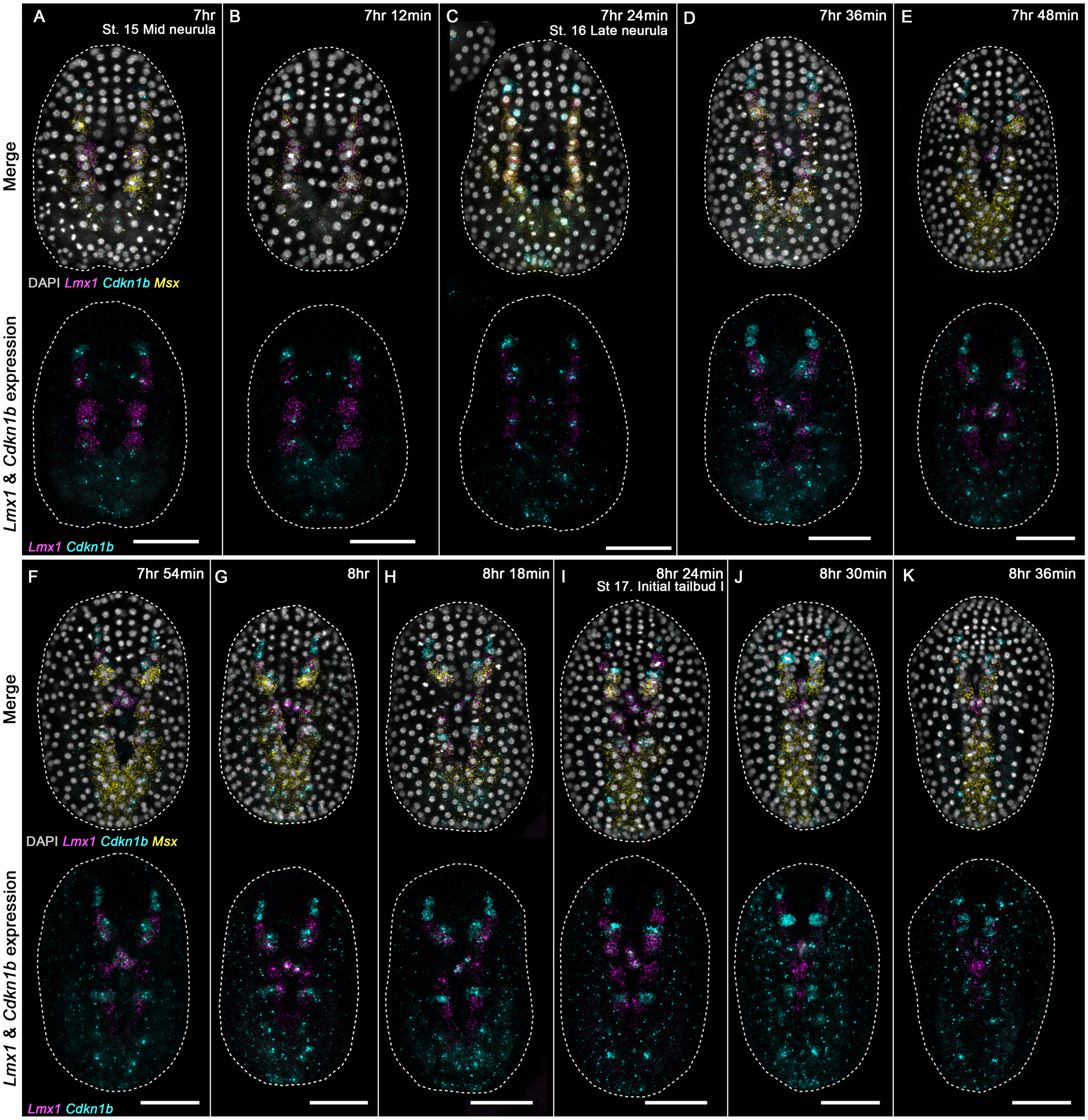

Figure S4: ***Lmx1*, *Msx*, and *Cdkn1b* expression patterns during neurulation.**
(A-K) Expression was examined throughout neural tube closure (∼7-8.5 hfp; samples collected every ∼6 minutes; 13 time points) via HCR *in situ* hybridization. The photographs are maximum-intensity projections of Z-projected image stacks overlaid in pseudocolor with HCR signals for *Lmx1* probe (magenta), *Msx* probe (yellow), and *Cdkn1b* probe (cyan). Nuclei were stained with DAPI (gray). Developmental stages are indicated in the photographs. Brightness and contrast of images was adjusted linearly. Numbers of embryos examined n = 15 per time point over n = 3 experiments. Scale bars, 50 µm.
